# A deep learning fusion network trained with medical records and laryngoscopic images in the early diagnosis of glottic carcinoma

**DOI:** 10.1016/j.isci.2025.114231

**Published:** 2025-11-26

**Authors:** Yi Shuai, Yun Li, Zhaohui Jin, Yuanyuan Li, Lin Chen, Wenqing Chen, Zhenqing Chen, Weixiong Chen, Ruxin Wang, Xiaomao Fan, Wenbin Lei

**Affiliations:** 1 Otorhinolaryngology Hospital, The First Affiliated Hospital, Sun Yat-sen University, Guangzhou, Guangdong, China; 2 College of Big Data and Internet, Shenzhen Technology University, Shenzhen, Guangdong, China; 3 Department of Otolaryngology-Head and Neck Surgery, The First People’s Hospital of Zhaoqing, Zhaoqing, Guangdong, China; 4 Department of Otolaryngology-Head and Neck Surgery, The First People’s Hospital of Foshan, Foshan, Guangdong, China; 5 Shenzhen Institute of Advanced Technology, Chinese Academy of Sciences, Shenzhen, Guangdong, China

**Keywords:** Oncology, Artificial intelligence

## Abstract

Early diagnosis of glottic carcinoma is crucial for improving the therapeutic outcomes of patients. This study aims to develop a deep learning fusion network that integrates analysis of structured medical records with laryngoscopic images to enable early and accurate diagnosis of glottic carcinoma. The model was trained and validated on data from a tertiary hospital in China. External validation was subsequently conducted across another two independent medical centers. Monomodal reference models were also developed for comparative analysis. To benchmark clinical utility, a human-machine adversarial cohort was constructed to enable direct performance comparisons between the model and human raters. Diagnostic accuracy was quantified using the area under the receiver operating characteristic curve (AUC). The model achieved superior diagnostic performance compared to monomodal models and achieved performance comparable to senior otolaryngologists. VLMN holds significant potential to reduce diagnostic delays and improve patient prognosis, particularly for junior otolaryngologists or in medically underserved areas.

## Highlights

• A deep learning fusion network combining medical records and laryngoscopic images
• The fusion model improves early diagnosis of glottic carcinoma
• The fusion model has the potential to be a powerful diagnostic tool for clinicians

## Graphical abstract

**Figure:**
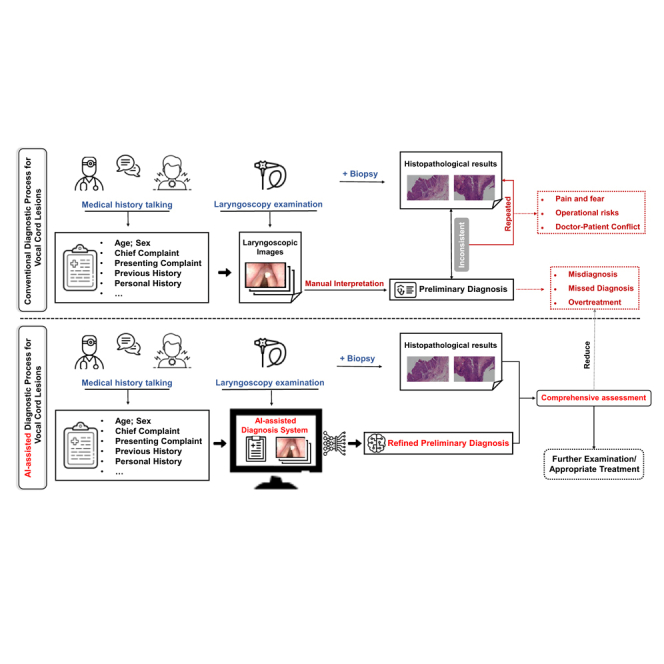

## Introduction

Laryngeal cancer is a common malignant tumor in the head and neck region, posing a significant burden on public healthcare service.^1^ According to GLOBOCAN, China reported 29,500 new cases and 16,900 deaths from laryngeal cancer in 2022.^2^ Glottic carcinoma is the main type of laryngeal cancer, accounting for over 70% of cases.^1^ Due to the specific anatomical features, glottic carcinoma can manifest symptoms such as hoarseness and globus sensation in the early stage, while these symptoms are nonspecific and may lead to delayed diagnosis. Consequently, less than 40% of patients can be accurately diagnosed in the early stage.^3^ Patients with advanced-stage disease often experience impairments in essential physiological functions—such as phonation, swallowing, and respiration—resulting in profound declines in the quality of life.^4^^,^^5^^,^^6^^,^^7^ Therefore, early diagnosis and intervention in glottic carcinoma are critical to improving the local disease control and long-term survival.^8^^,^^9^

The absence of specific diagnostic biomarkers has limited the development of effective screening strategies for glottic carcinoma. Currently, initial diagnosis typically relies on medical history assessment combined with laryngoscopic examination. However, the current diagnostic paradigm presents notable limitations, making it impractical for large-scale screening. First, the nonspecific symptoms complicate early diagnosis. Second, laryngoscopy faces challenges in differentiating vocal cord dysplasia from early glottic carcinoma due to their similar endoscopic features.^10^^,^^11^^,^^12^^,^^13^ Third, overlying “leukoplakia-like” mucosal changes may obscure the submucosal vascular patterns visible under narrow-band imaging (NBI), increasing diagnostic uncertainty.^14^ Compounding these issues are disparities in healthcare resource distribution and clinician expertise, which undermine the reliability of laryngoscopy-based early detection of glottic carcinoma.

Deep learning (DL) has demonstrated remarkable efficacy in lesion detection and oncological diagnostics. Recent studies in multimodal fusion techniques suggest that integrating heterogeneous data sources can substantially improve diagnostic accuracy across various cancer types.^15^^,^^16^^,^^17^ Despite these developments, current AI models for laryngeal cancer remain predominantly unimodal, relying solely on laryngoscopic images or videos.^18^^,^^19^^,^^20^ These monomodal models might overlook the temporal dynamics embedded in medical records, such as the progressive evolution of symptoms and the timeline of risk exposures. Additionally, they may fail to capture the latent associations with malignancy, such as immunosuppressive states and environmental exposure history, thereby limiting the clinical utility and reliability of models.

To address these gaps, we propose the development of a multimodal DL model that synergizes medical records with laryngoscopic imaging to improve diagnostic accuracy for early glottic carcinoma. We hypothesize that this approach will achieve high accuracy in distinguishing early glottic carcinoma from vocal cord dysplasia, reduce diagnostic delays (missed and misdiagnoses), minimize unnecessary invasive procedures, and ultimately enhance patient outcomes through timely intervention.

## Results

### Characteristics of patients and lesions

**Figure 1:**
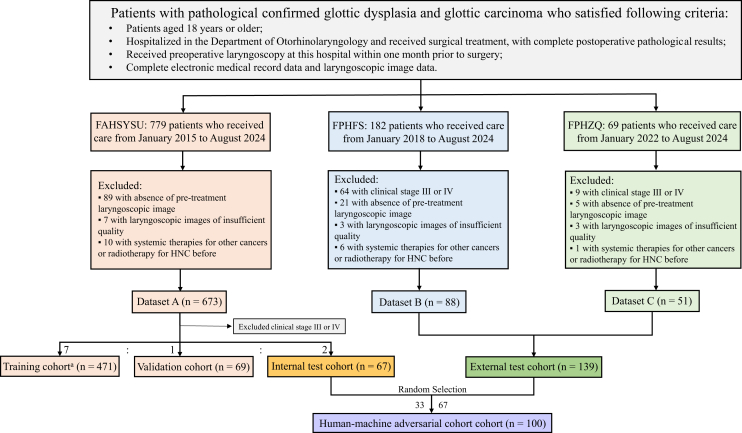
Recruitment flowchart for patients in this study FAHSYSU, the First Affiliated Hospital of Sun Yat-sen University; FPHFS, the First People’s Hospital of Foshan; FPHZQ, the First People’s Hospital of Zhaoqing. Training cohort^a^: the training cohort included advanced glottic carcinoma cases with T3 and T4 stages.

As shown in Table 1, a total of 471 patients (mean age, 61 · 64 years ±9 · 73; range, 35–91 years) from FAHSYSU were included in the training cohort, 69 patients (mean age, 61 · 19 years ±9 · 87; range, 38–89 years) in the validation cohort, and 67 patients (mean age, 59 · 58 years ±10 · 86; range, 33–78 years) in the internal test cohort. For the external test cohort, a total of 139 patients (mean age, 61 · 46 years ±12 · 03; range, 27–87 years) were enrolled from the other two hospitals. Patients with T3 or T4 stage were excluded from the validation cohort and test cohorts to focus on distinguishing early-stage glottic carcinoma from vocal cord dysplasia. The human-machine adversarial cohort comprised 100 patients (mean age, 59 · 92years ±12 · 97; range, 27–77 years) selected from the internal and external test cohorts. The patient recruitment workflow and dataset distribution are summarized in Figure 1. Most of the enrolled patients are males (Table 1), consistent with previous studies. As established risk factors, smoking and alcohol consumption were included for consideration. Detailed results of the pathological diagnosis and clinical stage are described in Table 1.

**Table 1:** The clinical characteristics of patients with glottic lesions

Clinical Characteristics	The First Affiliated Hospital of Sun Yat-sen University *n* = 673			External Test cohort *n* = 139		Human-Machine Adversarial cohort *n* = 100	
	Training cohort	Validation cohort^a^	Internal Test cohort^b^	The First People’s Hospital of Foshan	The First People’s Hospital of Zhaoqing	Internal Test cohort	External Test cohort
Patient Demographics							
No. of unique individuals	471	69	67	88	51	33	67
Age (mean ± SD, y)	61.64 ± 9.73	61.19 ± 9.87	59.58 ± 10.86	36.22 ± 10.78	58.43 ± 13.51	57.33 ± 11.81	61.19 ± 13.41
Sex (n, %)							
Male	449 (95.3%)	67 (97.1%)	64 (95.5%)	86 (97.7%)	46 (90.2%)	32 (97.0%)	64 (95.5%)
Female	22 (4.7%)	2 (2.9%)	3 (4.5%)	2 (2.3%)	5 (9.8%)	1 (3.0%)	3 (4.5%)
Cigarette (n, %)							
Yes	357 (75.8%)	56 (81.2%)	43 (64.2%)	64 (72.7%)	35 (68.6%)	20 (60.6%)	51 (76.1%)
No	114 (24.2%)	13 (18.8%)	24 (35.8%)	24 (27.3%)	16 (31.4%)	13 (39.4%)	16 (23.9%)
Alcohol (n, %)							
Yes	179 (38.0%)	26 (37.7%)	18 (26.9%)	27 (30.7%)	11 (21.6%)	10 (30.3%)	16 (23.9%)
No	292 (62.0%)	43 (62.3%)	49 (73.1%)	61 (69.3%)	40 (78.4%)	23 (69.7%)	51 (76.1%)
Pathological Diagnosis (n, %)							
Dysplasia	155 (32.91%)	32 (46.38%)	35 (52.24%)	18 (20.22%)	26 (50.98%)	17 (51.52%)	22 (32.84%)
Mild Dysplasia	50 (32.26%)	12 (37.50%)	10 (28.57%)	12 (66.67%)	17 (65.38%)	7 (41.18%)	14 (63.64%)
Moderate Dysplasia	57 (36.77%)	11 (34.38%)	7 (20.00%)	3 (16.67%)	5 (19.23%)	4 (23.53%)	4 (18.18%)
Severe Dysplasia	48 (30.97%)	9 (28.13%)	18 (51.43%)	3 (16.67%)	4 (15.38%)	6 (35.29%)	4 (18.18%)
Carcinoma	316 (67.09%)	37 (53.62%)	32 (47.76%)	71 (79.78%)	25 (49.02%)	16 (48.48%)	45 (67.16%)
Pathological T Stage of Glottic Carcinoma (n, %)							
T1	95 (30.06%)	19 (51.35%)	22 (68.75%)	53 (74.65%)	18 (72.00%)	11 (68.75%)	31 (68.89%)
T2	100 (31.65%)	18 (48.65%)	10 (31.25%)	18 (25.35%)	7 (28.00%)	5 (31.25%)	14 (31.11%)
T3	76 (24.05%)	0 (0.00%)	0 (0.00%)	0 (0.00%)	0 (0.00%)	0 (0.00%)	0 (0.00%)
T4	45 (14.24%)	0 (0.00%)	0 (0.00%)	0 (0.00%)	0 (0.00%)	0 (0.00%)	0 (0.00%)
Clinical Stage of Glottic Carcinoma (n, %)							
I	95 (30.06%)	19 (51.35%)	22 (68.75%)	53 (74.65%)	18 (72.00%)	11 (68.75%)	31 (68.89%)
II	100 (31.65%)	18 (48.65%)	10 (31.25%)	18 (25.35%)	7 (28.00%)	5 (31.25%)	14 (31.11%)
III	63 (19.94%)	0 (0.00%)	0 (0.00%)	0 (0.00%)	0 (0.00%)	0 (0.00%)	0 (0.00%)
IV	58 (18.35%)	0 (0.00%)	0 (0.00%)	0 (0.00%)	0 (0.00%)	0 (0.00%)	0 (0.00%)

The advanced cases were excluded from the validation cohort^a^ and the internal test cohort^b^, so the sum of the training, validation, and internal test cohorts is less than 673.

SD, standard deviation.

### Diagnostic performance of deep learning models

**Figure 2:**
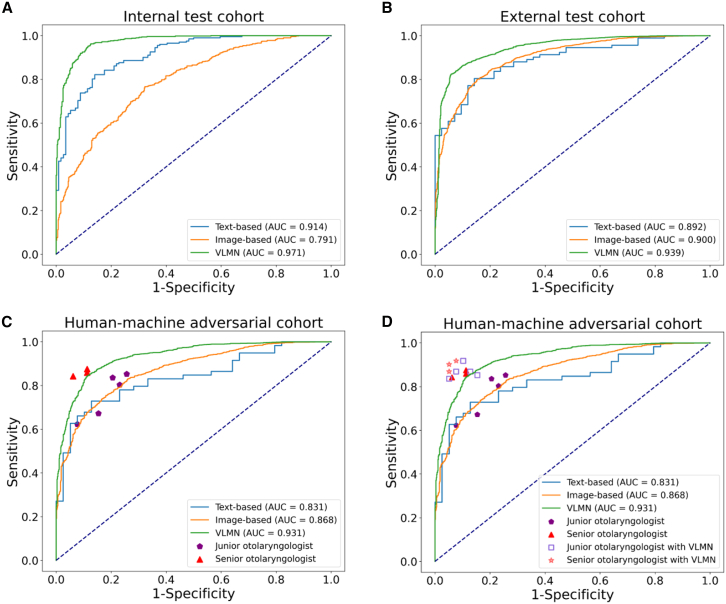
ROC curve of text-based monomodal model, image-based monomodal model, VLMN model, and human raters in the evaluation cohorts (A) ROC curves of text-based monomodal model, image-based monomodal model, and VLMN model in the internal test cohorts. (B) ROC curves of text-based monomodal model, image-based monomodal model, and VLMN model in the external test cohorts. (C) ROC curves of text-based monomodal model, image-based monomodal model, VLMN model, and human raters in the human-machine adversarial cohort. (D) ROC curves of monomodal models, VLMN model, human raters alone, and human raters with VLMN in the human-machine adversarial cohort. ROC, receiver operating characteristic; AUC, area under the ROC curve. VLMN, vision large language model based multimodal fusion network model.

**Figure 3:**
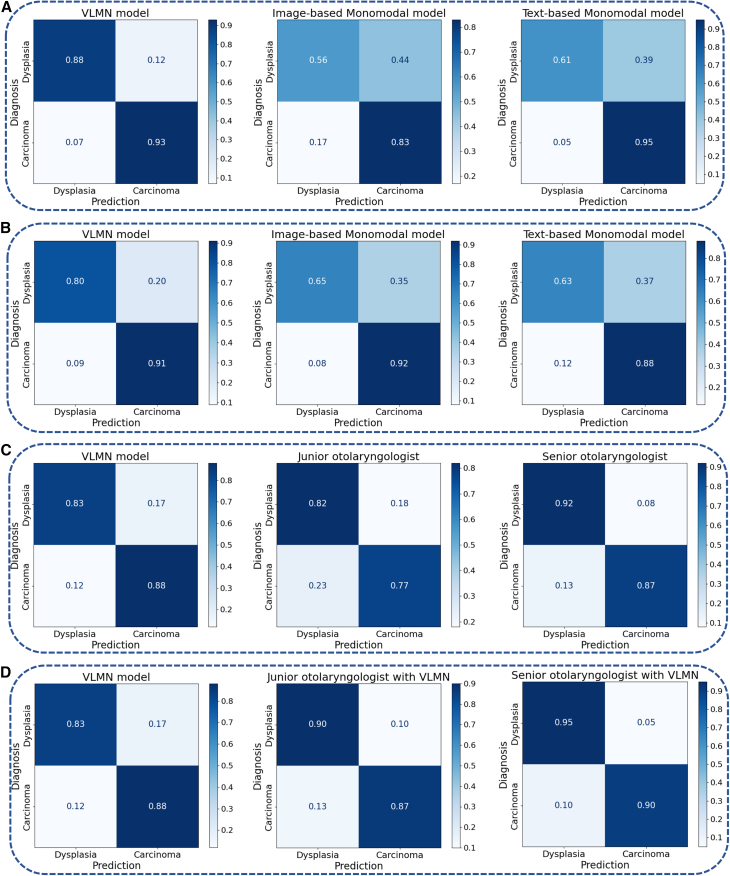
Confusion matrices of DL models and human raters in the evaluation cohorts (A) Confusion matrices of text-based monomodal model, image-based monomodal model, and VLMN model in the internal test cohorts. (B) Confusion matrices of text-based monomodal model, image-based monomodal model, and VLMN model in the external test cohorts. (C) Confusion matrices of the VLMN model and human raters in the human-machine adversarial cohort. (D) Confusion matrices of the VLMN model and human raters with VLMN in the human-machine adversarial cohort. VLMN, vision large language model based multimodal fusion network model.

When evaluated in the internal test cohort, the diagnostic performance of the VLMN model was better than both of the monomodal DL models. In comparison to the monomodal DL model with medical records alone, the AUC of the VLMN model integrating analysis of medical records and laryngosopic images was significantly higher (0 · 971; 95% confidence interval [CI], 0 · 968–0·974 vs. 0·914; 95% CI, 0·913-0·915; *p* = 0·035) (Figure 2; Table 2). The same result was observed when comparing to the monomodal DL model with laryngoscopic images alone (0·971; 95% CI, 0·968-0·974 vs. 0·791; 95% CI, 0·788-0·793; *p* < 0·0001) (Figure 2; Table 2). Moreover, the VLMN showed accuracy, sensitivity, and specificity, positive predictive value (PPV), and negative predictive value (NPV) surpassing the monomodal models (Table 2). The percentage of correct classification of DL models was presented in the confusion matrix. Overall, the DL models performed better in diagnosing carcinoma than dysplasia cases (Figure 3), especially for the text-based model. However, the percentage of correct classification of the VLMN model in dysplastic lesions was remarkably increased compared with the monomodal models (88% vs. 56% for the image-based model; 88% vs. 61% for the text-based model) (Figure 3).

**Table 2:** Diagnostic performance of different DL models and human raters on the test cohorts

Ratings	Accuracy (95% CI)	AUC (95% CI)	Sensitivity (95% CI)	Specificity (95% CI)	PPV (95% CI)	NPV (95% CI)
**Internal test cohort**						

VLMN model	0.901 (0.888–0.914)	0.971 (0.968–0.974)	0.858 (0.824–0.891)	0.956 (0.931–0.981)	0.959 (0.932–0.985)	0.845 (0.797–0.892)
Image-based monomodal model	0.696 (0.683–0.709)	0.791 (0.788–0.793)	0.649 (0.628–0.670)	0.779 (0.758–0.801)	0.834 (0.799–0.869)	0.562 (0.506–0.619)
Text-based monomodal model	0.829 (0.820–0.837)	0.914 (0.913–0.915)	0.813 (0.809–0.817)	0.875 (0.844–0.907)	0.951 (0.935–0.966)	0.612 (0.600–0.624)

**External test cohort**						

VLMN model	0.884 (0.875–0.893)	0.939 (0.933–0.944)	0.945 (0.930–0.960)	0.682 (0.638–0.726)	0.908 (0.884–0.933)	0.784 (0.710–0.858)
Image-based monomodal model	0.863 (0.856–0.870)	0.900 (0.899–0.901)	0.913 (0.902–0.924)	0.659 (0.623–0.695)	0.916 (0.895–0.937)	0.648 (0.593–0.703)
Text-based monomodal model	0.803 (0.792–0.814)	0.892 (0.883–0.902)	0.844 (0.793–0.894)	0.716 (0.660–0.773)	0.880 (0.816–0.945)	0.633 (0.485–0.782)

**Human-machine adversarial cohort**						

VLMN model	0.870 (0.865–0.875)	0.931 (0.930–0.933)	0.939 (0.927–0.951)	0.709 (0.667–0.750)	0.883 (0.875–0.890)	0.834 (0.828–0.841)
Image-based monomodal model	0.806 (0.795–0.818)	0.868 (0.864–0.872)	0.853 (0.831–0.876)	0.683 (0.632–0.734)	0.880 (0.831–0.929)	0.624 (0.542–0.706)
Text-based monomodal model	0.718 (0.682–0.755)	0.831 (0.826–0.836)	0.742 (0.671–0.812)	0.682 (0.665–0.700)	0.830 (0.769–0.891)	0.549 (0.371–0.727)
Junior otolaryngologist	0.780 (0.741–0.816)	–	0.757 (0.705–0.804)	0.815 (0.754–0.867)	0.865 (0.818–0.904)	0.682(0.618–0.742)
Senior otolaryngologist	0.887 (0.845–0.920)	–	0.869 (0.811–0.914)	0.915 (0.848–0.958)	0.949 (0.894–0.971)	0.817 (0.740–0.879)
Junior otolaryngologist with VLMN	0.880 (0.848–0.907)	–	0.869 (0.826–0.901)	0.897 (0.846–0.936	0.930 (0.894–0.957)	0.814 (0.755–0.864)
Senior otolaryngologist with VLMN	0.913 (0.876–0.943)	–	0.896 (0.843–0.936)	0.940 (0.881–0.976)	0.959 (0.918–0.983)	0.853 (0.780–0.901)

VLMN, vision large language model based multimodal fusion network; AUC, area under the receiver operating characteristic curve; PPV, positive predictive value; NPV, negative predictive value; CI, confidence interval; NA, not applicable.

When evaluated in the external test cohort, the VLMN model remained superior to both of the monomodal models. Compared to the monomodal DL model based on medical records alone, the VLMN model had a significantly higher AUC (0·939; 95% CI, 0·933-0·944 vs. 0·892; 95% CI, 0·883-0·902; *p* = 0·042). Similarly, a comparable result was found in the monomodal DL model with only laryngoscopic images (0·939; 95% CI, 0·933-0·944 vs. 0·900; 95% CI, 0·899-0·901; *p* < 0·0001). Furthermore, the VLMN had a better accuracy, sensitivity, and NPV than the monomodal models (Table 2). As shown in the confusion matrix, all the DL models outperformed in diagnosing carcinoma than dysplasia cases (Figure 3). However, the VLMN model could reduce the risk that the monomodal DL models might misclassify some dysplastic lesions as carcinoma.

**Figure 4:**
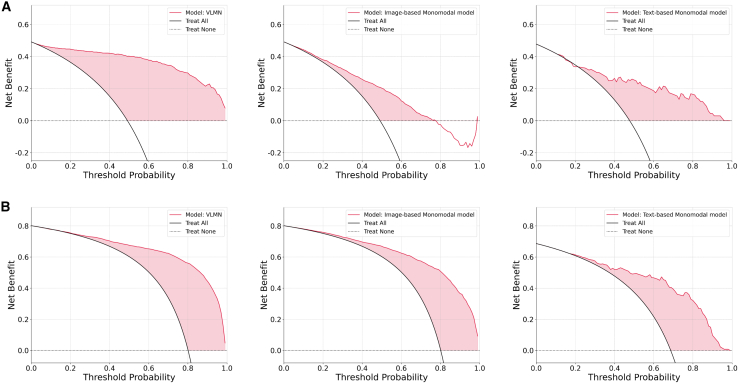
DCA curve of the VLMN model, image-based monomodal model, and text-based monomodal model in the test cohorts (A) DCA curves of the VLMN model, image-based monomodal model, and text-based monomodal model in the internal test cohorts. (B) DCA curves of the VLMN model, image-based monomodal model, and text-based monomodal model in the external test cohorts. DCA, decision curve analysis curve; VLMN, vision large language model based multimodal fusion network model.

Moreover, we use decision curve analysis (DCA) curves to evaluate the net benefit of the VLMN model in clinical practice. As shown in the Figure 4, at threshold probabilities exceeding 30%, the VLMN model was markedly superior to both the “Treat All” and “Treat None” strategies, demonstrating that its clinical application within this interval could substantially decrease the rate of unnecessary interventions while maintaining the robust identification of high-risk patients. However, at threshold probability range below 20%, the DCA curve of the VLMN model almost converged with the “Treat All” strategy, rendering its predictive utility limited within this range. Additionally, regardless of whether in the internal or external test cohort, the DCA curve of the VLMN model was superior to both of the monomodal models, indicating its higher benefit for clinical decision-making.

To test our hypothesis – that the inclusion of advanced-stage (T3/T4) cases during training would improve the model’s overall classification performance – we conducted an additional experiment. The results showed that the model trained on all enrolled (T1-T4) cases achieved a higher accuracy than the model trained exclusively on early-stage (T1-T2) cases (Table S4, Figures S2 and S3).

Additionally, we further analyzed the performance of VLMN on the WLI and NBI images, respectively. The results demonstrated that the VLMN model achieved robust diagnostic performance using either WLI or NBI images in both internal and external test cohorts (Table S5, Figure S4, and S5). Notably, its reliable performance with WLI alone highlights its applicability in medically underserved areas where NBI is unavailable. Furthermore, the VLMN model exhibited better performance on WLI images compared to NBI images. However, the highest diagnostic accuracy was consistently obtained when all image modalities were combined. Several factors may contribute to these findings. First, the inclusion of NBI images enhances submucosal vascular visualization, which is known to facilitate early cancer detection. Second, our review of the enrolled NBI images revealed that they were frequently captured at a much closer distance, resulting in an enlarged view of the local lesion area to better visualize intrapapillary capillary loops (IPCLs). While this approach improves the clarity of local microvascular features, it may also compromise the ability to assess the overall extent of the lesion comprehensively. The proportion of WLI and NBI images used in training, validation and test cohorts was shown in the Supplementary material (Table S3).

To explore the potential factors that affect the diagnostic accuracy in the external test cohort, we contrasted the performance of the VLMN model in the two hospitals enrolled in the external test cohort, respectively. The VLMN model had a higher accuracy on the dataset from FPHFS than FPHZQ (0·905; 95% CI, 0·897–0·913 vs. 0·708; 95% CI, 0·684–0·733). The diagnostic performance of the VLMN model in the sub-centers is presented in Table S2. By comparing the original data from two sub-centers and corresponding visualizations, we have summarized the following factors that may interfere with the diagnostic performance. First, the FPHFS cohort included a larger number of cases and, on average, more laryngoscopic images per case compared to the FPHZQ cohort (Table S3). Second, the overall image quality in FPHFS was markedly higher, providing clearer visual cues. Third, laryngoscopic images from FPHFS included white-light imaging (WLI) and a substantial number of narrow-band imaging (NBI) images, whereas the FPHZQ cohort contained NBI images in only a subset of cases. To better understand the inferior image quality observed in FPHZQ compared to FPHFS, we considered contributing factors at the equipment, technical, and economic levels. Device selection is often closely tied to the local economic conditions. Moreover, the technical proficiency of endoscopists performing laryngoscopy is also associated with regional socioeconomic status. As shown in Table S1, the laryngoscopy devices used at FPHZQ were comparatively outdated, likely reflecting local economic constraints. A comparison between FPHFS and FAHSYSU further supports this interpretation, as both centers employed similar types of equipment and demonstrated comparable diagnostic accuracy on their respective test cohorts. Nevertheless, the model maintained robust diagnostic performance at FPHZQ, suggesting potential applicability even in economically under-resourced settings. This finding is consistent with our original intention of leveraging AI to facilitate early diagnosis of glottic carcinoma in medically underserved areas.

### Diagnostic performance of deep learning models vs. human raters

When evaluated in the human-machine adversarial cohort, the VLMN model demonstrated a significant higher accuracy compared to the junior otolaryngologists (mean accuracy: 0 · 870 vs. 0 · 780; *p* = 0 · 038), and performed at a level comparable to the senior otolaryngologists (mean accuracy: 0 · 870 vs. 0 · 887; *p* = 0 · 72) (Table 2). The same observation was made in sensitivity and NPV (Table 2). Additionally, as shown in the confusion matrix (Figure 3), the VLMN model had a higher percentage of correct classification for glottic carcinoma than human raters. These results highlighted that VLMN provided superior diagnostic advantages in distinguishing early glottic carcinoma from vocal cord dysplasia compared to junior otolaryngologists. However, human raters still maintained a substantial advantage in diagnosing vocal cord dysplasia. When diagnosing with the assistance of VLMN, the junior otolaryngologists demonstrated significantly improved diagnostic accuracy, surpassing the performance of VLMN alone (mean accuracy: 0·880 vs. 0·870; *p* = 0·72) and approaching that of senior otolaryngologists (mean accuracy: 0·880 vs. 0·887; *p* = 0·91) (Table 2).

### Visual evidence of the vision large language model-based multimodal fusion network model

**Figure 5:**
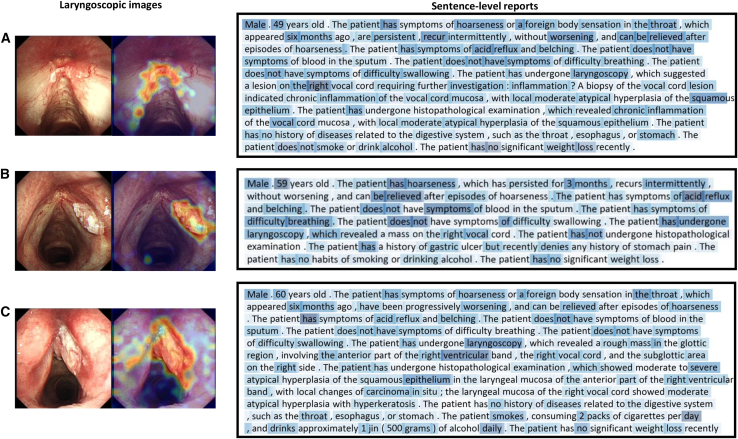
Representative heat maps of the VLMN model’s analysis using laryngoscopic images and the sentence-level reports extracted from medical record text The varying colors reflect different levels that the VLMN model concentrates on. For the laryngoscopic images, a redder color on the heatmap indicates a greater contribution of that region’s features to the model’s prediction. The sentence-level reports are standardized reports extracted from medical record text using prompts based on the Llama large model; a darker blue on the heatmap indicates closer attention of the model. (A) The heat maps of the laryngoscopic image and the sentence-level report from a patient diagnosed with vocal cord dysplasia. (B) The heat maps of the laryngoscopic image and the sentence-level report from a patient diagnosed with T1-stage glottic carcinoma. (C) The heat maps of laryngoscopic image and sentence-level report from a patient diagnosed with T2-stage glottic carcinoma. VLMN, vision large language model based multimodal fusion network model.

To provide a more intuitive representation of the model’s predictions, we conducted visualization analyses of laryngoscopic images and medical record texts separately. In the image visualization, the color gradient of the heatmap reflects the model’s attention distribution, with warmer colors corresponding to higher response—those that contribute most significantly to the prediction. As shown in Figure 5, the high-response regions in the heatmaps align closely with the lesion areas in the laryngoscopic images, suggesting that the model can accurately locate the lesion and capture key features. In the text visualization, the color intensity reflects the model’s level of attention, with darker shades indicating higher focus. Figure 5 demonstrates that the VLMN model primarily focuses on critical terms related to the disease (e.g., hoarseness, difficulty breathing, laryngoscopy, and so forth), indicating that the model can effectively identify key words in the medical record text. The visualization results enhance the interpretability and credibility of the model, fostering trust in the AI-assisted system among clinicians and improving its clinical applicability. The representative heatmaps of the DL models for various glottic lesions are shown in Figure 5.

## Discussion

**Figure 6:**
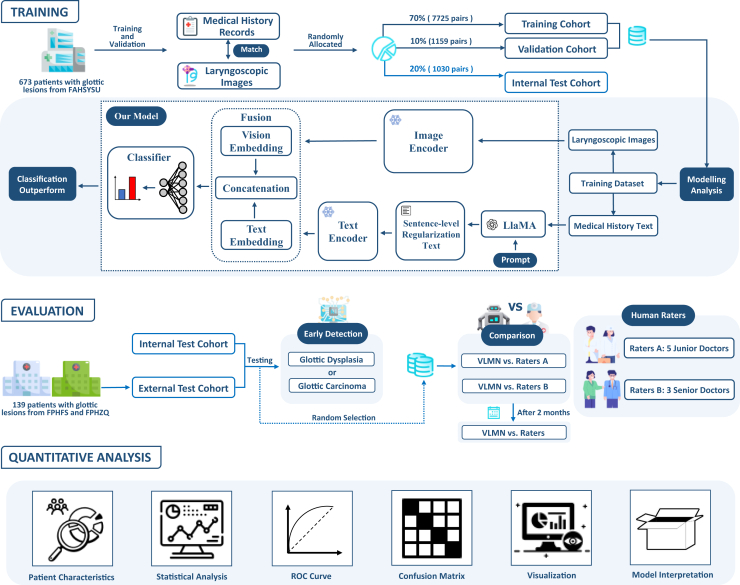
Overview of the study design FAHSYSU, the First Hospital Affiliated to Sun Yat-sen University; FPHFS, the First People’s Hospital of Foshan; FPHZQ, the First People’s Hospital of Zhaoqing; LlaMA, large language model meta AI; VLMN, vision large language model based multimodal fusion network model; ROC, receiver operating characteristic.

In this ‌retrospective multicenter study‌, we designed and validated a ‌multimodal DL framework‌—the ‌VLMN model‌—to enhance the early diagnosis of glottic carcinoma and benchmark its performance against otolaryngologists. The study design is summarized in Figure 6. Our results demonstrated that the VLMN model, which synergistically integrated ‌structured medical records‌ and ‌laryngoscopic images‌, achieved ‌superior diagnostic accuracy‌ compared to monomodal models (*p* < 0 · 05). Moreover, the VLMN model outperformed junior otolaryngologists in discriminating early glottic carcinoma from vocal cord dysplasia and achieved ‌diagnostic parity‌ with senior otolaryngologists. Notably, VLMN-assisted diagnosis of early glottic carcinoma exhibited significantly better performance among junior otolaryngologists, surpassing that of VLMN alone and approaching senior otolaryngologists. These findings support the VLMN model as a ‌clinically viable tool‌ for enhancing early detection rates, particularly for less experienced otolaryngologists.

Medical records and laryngoscopic images are indispensable for diagnosing glottic carcinoma. The medical history data extracted from electronic medical records (EMRs) provides a detailed account of clinical information, such as symptoms, disease progression, visit history, and risk factors (e.g., laryngopharyngeal reflux, smoking, alcohol consumption, and genetic predisposition).^21^^,^^22^ Meanwhile, laryngoscopy enables the direct observation of lesion morphology and vocal cord mobility, offering diverse visual information such as lesion size, location, extent and surface characteristics.^1^^,^^23^ When augmented with NBI, it can enhance the detection of early malignancies by assessing intraepithelial papillary capillary loops (IPCLs).^14^^,^^24^^,^^25^ The fusion of two modalities facilitates a more comprehensive assessment of lesions, thereby enhancing the diagnostic accuracy. By utilizing a multimodal DL model, we aim to improve the diagnostic accuracy of early glottic carcinoma, especially for inexperienced otolaryngologists and regions with limited medical resources.

**Figure 7:**
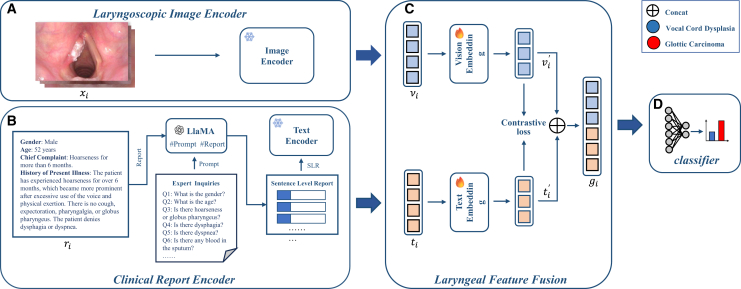
Detailed workflow for the data processing and the development of the VLMN model (A) The vision transform (Vit) model for extracting features from the laryngoscope images. (B) The module for generating sentence-level reports and extracting features from clinical reports. (C) The module for the fusion of multimodal features. (D) The module classify fusion features.

Most existing studies have focused on a dichotomous task or classification of a few preselected laryngeal diseases. For example, Li Y et al. applied a DL model to distinguish benign from malignant laryngeal lesions.^26^ Zhao Q et al. reported that the performance of DL models can match clinicians in the classification of laryngeal lesions.^27^ However, due to the morphological and clinical similarities between early glottic carcinoma and vocal cord dysplasia—particularly under laryngoscopic examination—these models may struggle to differentiate them. Additionally, most previous studies have adopted single-modality approaches,^18^^,^^19^^,^^20^ which present limited information.^28^ Although some existing models, such as LA-ViT, ViT-AMC, and FDTs, have achieved promising results on unimodal data (e.g., laryngoscopic or histopathological images) by leveraging advanced attention mechanisms and multi-objective optimization strategies,^29^^,^^30^^,^^31^ they are still limited to a single modality and do not incorporate textual clinical information. In contrast, our work explores a multimodal transformer-based framework that integrates laryngoscopic images with medical records for glottic carcinoma diagnosis. This approach not only captures morphological patterns from images but also leverages temporal and semantic cues embedded in the medical history, thereby providing a more comprehensive approach aligned with clinical practice. Moreover, for the text modality, we selected only a portion of the medical history from the admission records. These records can be easily gathered through a clinical interview, making it well-suited for the early detection of disease. For the image modality, some cases in our datasets only had white light images, with NBI images absent. As a result, our model remains effective in such scenarios, especially in regions with limited access to NBI technology. Additionally, by using a pre-trained model for image preprocessing, we eliminated the need for manual region of interest (ROI) annotations, significantly reducing labor costs. The detailed workflow of VLMN is shown in Figure 7.

When contrasting the performance of two monomodal models, we found that the text-based model outperformed the image-based model in the internal test cohorts. While in the external test cohort, the image-based model performed better than the text-based model. Considering the visualization results, we propose that the following factors might explain. As shown in Figure 5, the visit history in HPI plays a vital role in the text modality. Notably, before visiting a tertiary hospital (such as FAHSYSU), a few patients from the remote regions may have undergone laryngoscopy-guided biopsy at local hospitals (such as FPHFS or FPHZQ). The biopsy pathological results from external hospitals are often described in the visit history, which may enhance the model’s diagnostic performance, particularly by offering guidance in cases with biopsy-proven malignancies from external institutions. As a result, in the internal cohort (FAHSYSU), the text modality may provide much more valuable information compared to the image modality. However, in the external cohort (FPHFS and FPHZQ), the pathological information in the text modality was nearly absent, making the image modality of greater diagnostic value.

During pathological verification, we observed several cases in which biopsy indicated dysplasia, whereas postoperative pathology confirmed carcinoma. The discrepancy highlights the inherent limitations of preoperative biopsy pathology, particularly in early-stage glottic carcinoma. First, biopsies are often performed under topical anesthesia, and vocal cord motion can impair sampling accuracy. Second, cancerous lesions exhibit spatiotemporal heterogeneity, with some cancer nests occupying as little as 12–15% of the affected area, thereby limiting lesion accessibility. Given the limited sampling area of a 3.2 mm biopsy forceps, microinvasive foci may be missed.^32^ Third, Sampling quality is influenced by the technical expertise of the laryngoscopist. Consequently, biopsy errors are sometimes inevitable in clinical practice, which may cause diagnostic delays. In contrast, by integrating multimodal information, the VLMN model enables more comprehensive and accurate diagnostic assessments, thereby reducing overdependence on biopsy results, mitigating diagnostic uncertainty or misjudgments due to sampling errors, and potentially preventing delays in clinical decision-making.

In conclusion, we developed a multimodal fusion deep learning model (VLMN) that combined medical records and laryngoscopic images for differentiating early glottic carcinoma from vocal cord dysplasia. By providing ‌interpretable diagnostic support‌, the VLMN model can address critical gaps in the diagnostic pathway for glottic carcinoma and mitigate diagnostic delays to improve patient outcomes.

### Limitations of the study

Despite a satisfactory diagnostic performance for early glottic carcinoma of VLMN, this study has several limitations. First, the prospective validation was not conducted in our study. Future research could create a larger prospective cohort to further validate the performance of the model. Second, only the hospitalized and pathologically confirmed cases were enrolled. It may bring bias from the overrepresentation of malignant cases.^33^ However, we believe that the advantages of a diagnosis verified by pathology outweigh the potential bias. The subsequent research will prioritize clinical validation through prospective, multicenter trials to quantify the model’s utility in real-world scenarios, particularly the capacity to enhance early glottic carcinoma detection rates in medically underserved areas. Furthermore, we intend to expand our dataset with more granular pathological labels and refine the model to support more precise clinical decision-making. We also plan to incorporate a broader range of clinically meaningful prompts to comparatively assess their contributions to model performance—such as HPV status, occupational and environmental exposures, dietary habits, immune status, and laboratory data.^34^^,^^35^^,^^36^

## Resource availability

### Lead contact

Further information and requests for resources should be directed to and will be fulfilled by the lead contact, Lei Wenbin (E-mail: leiwb@mail.sysu.edu.cn).

### Materials availability

This study did not generate new unique reagents.

### Data and code availability

• Due to patient privacy, all the datasets generated or analyzed in this study are available from the lead contact upon reasonable request.
• The code for the model development has been deposited at GitHub (https://github.com/ZhaoHuiii/VLMN) and is publicly available as of the date of publication. The DOI is also listed in the key resources table.
• Any additional information required to reanalyze the data reported in this article is available from the lead contact upon reasonable request.

## Acknowledgments

This work was supported by the 10.13039/100016085Basic and Applied Research Foundation of Guangdong Province (No. 2022B1515130009), the 10.13039/100014718National Natural Science Foundation of China (No. 82473271, 62473267, 82403695 and 82273053), the 10.13039/501100019031Guangzhou Municipal Key Research and Development Program Fund (No. 2025B03J0019), the 5010 Clinical Research Program of 10.13039/501100002402Sun Yat-sen University (No. 2017004), and the 10.13039/100007219Natural Science Foundation of Top Talent of 10.13039/100019839SZTU (GDRC202318). The funders had no role in the study design, data collection, data analyses, interpretation, or writing of the report.

## Author contributions

Conceptualization: WB.L., XM.F., and Y.L.; data curation: Y.S., YY.L., L.C., ZQ.C., and WX.C.; formal analysis: ZH.J. and Y.S.; funding acquisition: WB.L. and XM.F.; investigation: Y.S., ZH.J., Y.L., YY.L., and WQ.C.; methodology: ZH.J., XM.F., and RX.W.; project administration: WB.L., XM.F., and Y.L.; resources: WB.L. and XM.F.; software: ZH.J. and Y.S.; supervision: WB.L., XM.F., and Y.L.; validation: Y.S., ZH.J., and Y.L.; visualization: Y.S. and ZH.J.; writing-original draft: Y.S. and ZH.J.; accessing and verifying the underlying data: XM.F., Y.L., YY.L., and WB.L.

## Declaration of interests

The authors XM.F., ZH.J., Y.S., Y.L., WB.L., and WQ.C. report a patent for No.2025103725379 pending.

## STAR★Methods

### Key resources table

**Table:** 

REAGENT or RESOURCE	SOURCE	IDENTIFIER
**Deposited data**		

Raw medical records of patients with glottic lesions	This paper	N/A
Raw laryngoscopic images of patients with glottic lesions	This paper	N/A
Code for model development	This paper	https://github.com/ZhaoHuiii/VLMN

**Software and algorithms**		

Python (version 3.9.0)	Python Software Foundation	https://www.python.org/
LlaMA large language model	Grattafiori A et al.^37^	https://doi.org/10.48550/arXiv.2407.21783
Pre-trained Vision Transformer model	Dosovitskiy A et al.^38^	https://doi.org/10.48550/arXiv.2010.11929

### Experimental model and study participant details

#### Ethics

This retrospective multicenter study was approved by the Ethics Committee of the First Affiliated Hospital of Sun Yat-sen University (Approval No. [2023]755-1). Informed consent was waived by the institutional review boards of all participating hospitals due to the study’s retrospective design. Reporting adhered to the Standards for Reporting of Diagnostic Accuracy Studies (STARD) guidelines.

#### Datasets and cohort design

Between January 1, 2015, and August 31, 2024, a retrospective cohort of 673 patients with histopathologically confirmed glottic lesions was enrolled at the First Affiliated Hospital of Sun Yat-sen University (FAHSYSU), Guangzhou, China. This cohort included 673 medical records and 10,715 laryngoscopic images, partitioned into training (70%, *n* = 471), validation (10%, *n* = 67), and internal test (20%, *n* = 135) cohorts. To assess generalizability, a multicenter external validation study was conducted across two Chinese hospitals with varying healthcare resource levels. From January 1, 2018, to August 31, 2024, 139 cases (139 medical records and 3,628 laryngoscopic images) were collected from the First People’s Hospital of Foshan (FPHFS) and the First People’s Hospital of Zhaoqing (FPHZQ), forming the external test cohort. To benchmark model performance against human raters, a human-machine adversarial cohort (*n* = 100, 2,617 images) was created by randomly sampling cases from internal and external test cohorts in a 1:2 ratio. Notably, advanced-stage glottic carcinoma cases (T3/T4) were included in the training cohort to enhance the model’s ability to learn malignancy-associated features, such as invasive growth patterns and vascular abnormalities. However, to rigorously evaluate early detection performance, these advanced cases were excluded from the validation and test cohorts, ensuring the assessment focused exclusively on differentiating early glottic carcinoma (T1/T2) from vocal cord dysplasia. To demonstrate the feasibility of our idea, we also conducted an experiment training the model exclusively on early-stage (T1/T2) cases and evaluated it on the same test cohorts. The patient recruitment workflow and dataset distribution are summarized in Figure 1.

Inclusion criteria were as follows: (1) Patients aged 18 years or older; (2) Hospitalized in the Department of Otorhinolaryngology between January 1, 2015 and August 31, 2024, and diagnosed as glottic carcinoma or vocal cord dysplasia based on postoperative pathology; (3) Received preoperative laryngoscopy in the hospital within one month prior to surgery; (4) Complete data of EMRs and laryngoscopic images. Exclusion criteria included: (1) Patients who had undergone relevant laryngeal surgery elsewhere (except tracheostomy) before visiting the sub-centers; (2) Patients with a history of chemotherapy, immunotherapy or targeted therapy for other cancers, or radiotherapy for head and neck cancers (HNC); (3) Patients with other head and neck cancers invading the glottis; (4) Glottic lesions occupying less than 5% of the laryngoscopic image or with severe architectural distortion; (5) Laryngoscopic images with blood, saliva, bubbles, biopsy forceps, or other interference affecting the overall view. It was strictly prohibited to have any overlap between the datasets used for training, validation, and testing purposes.

#### Pathological ground truth

The glottic lesions enrolled in this study were classified as glottic carcinoma or vocal cord dysplasia based on the postoperative histopathological results. According to the 8th edition of the TNM classification for head and neck tumors by the American Joint Committee on Cancer (AJCC) and the International Agency for Research on Cancer (IARC), early glottic carcinoma (EGC) is defined as T1aN0M0, T1bN0M0 (stage I), and T2N0M0 (stage II).^39^ The pathological types of vocal cord dysplasia include mild, moderate, and severe dysplasia.^40^^,^^41^

Important to note that, some patients in the datasets may have undergone one or more biopsies prior to surgery, including a few performed at external institutions. To confirm the classification of patients, all final diagnostic labels were manually verified based on postoperative histopathological results. At least two pathologists (≥5 years of experience) independently assessed tissue sections according to the WHO Classification of Tumors. The final diagnosis was established upon consensus between pathologists. For pathology results from other hospitals, patients were required to submit the original pathology slides for review, with the pathological consultation results from sub-centers serving as the reference standard. This approach was adopted to minimize potential diagnostic bias and interference on model training. During the process, we observed a phenomenon: for enrolled cases diagnosed as vocal cord dysplasia or leukoplakia upon discharge, the preoperative biopsy pathology did not always align with the postoperative pathology. We selected the highest-grade pathology classification on paraffin samples as the final diagnosis. Table 1 summarizes the frequencies of diagnoses in the training, validation, and test cohorts.

### Method details

#### Study design

This is a retrospective, multicenter study. We developed the Vision Large Language Model-based Multimodal Fusion Network (VLMN), a DL framework integrating medical records and laryngoscopic images for the early diagnosis of glottic carcinoma. Monomodal DL models—trained exclusively on either medical records or laryngoscopic images—were constructed for comparative analysis. Model performance was evaluated on internal and external test cohorts. A human-machine adversarial cohort was subsequently used to benchmark VLMN against human raters (junior and senior otolaryngologists). The study design is summarized in Figure 6.

#### Data acquisition

The medical records were exported from the hospitals’ electronic medical records (EMRs) system in TXT format. Each record was written by the assigned attending physician responsible for the patient, leading to some variability in the quality of the records. In this modality, we only extracted age, gender, chief complaint, history of present illness (HPI), past history and personal history from the admission records as input.

For all the hospitals, the laryngoscopic images were captured by endoscopists with at least five years of experience in laryngoscopy. Notably, both white light images (WLI) and narrow band imaging (NBI) images were enrolled in the datasets, but the NBI images were missing in some cases of external test cohort due to the limitation of resource. Nevertheless, the model trained and tested under these conditions can better reflect real-world scenarios. The manufacturers of laryngoscope equipment used in this study are listed in Table S1.

#### Data preprocessing

To address the inherent redundancy and heterogeneity in unstructured medical records, we implemented a prompt engineering framework guided by otolaryngologists.^42^ Clinically relevant sentences (e.g., symptom descriptions, smoking history, dysphonia duration) were extracted using domain-specific prompts, while non-diagnostic content (e.g., administrative notes, unrelated past medical history) was systematically filtered. This process generated standardized, sentence-level diagnostic summaries, minimizing noise and enhancing feature relevance for downstream model training.^37^ To further improve robustness, the order of sentences within these summaries was randomized during training, enforcing sequence invariance and reducing overfitting risks. The prompt template of LlaMA was shown in Figure S1.

Laryngoscopic images were preprocessed using a Vision Transformer (ViT)-compatible pipeline.^38^ Images were resized to 224 × 224 pixels, normalized to zero mean and unit variance (using ImageNet-derived parameters), and augmented with learnable positional encodings to preserve spatial relationships. Processed images were paired with their corresponding standardized medical summaries to create multimodal training units (image-text pairs), enabling joint representation learning across visual and textual modalities. Full preprocessing protocols, including hyperparameters and normalization details, are provided in Method S1.

#### Deep multimodal fusion network architecture

The Vision-Language Multimodal Network (VLMN) comprises four core components: (1) a ‌laryngoscope image encoder‌ that extracts visual embeddings from laryngoscopic images, (2) a ‌text encoder‌ that processes medical records into textual embeddings, (3) a ‌multimodal feature fusion network‌ that integrates these embeddings into a unified feature vector, and (4) a ‌classifier‌ that predicts diagnostic outcomes. The detailed workflow of VLMN is shown in Figure 7. The fusion network aligns cross-modal features to capture complementary anatomical and clinical insights, enabling robust laryngeal cancer diagnosis. Model architecture details are provided in Method S2.

We employed ‌cross-entropy loss‌ for its computational efficiency and effectiveness in minimizing prediction-label discrepancies. To address cross-modal heterogeneity between imaging and textual data, ‌contrastive loss‌ was introduced,^43^ reducing inter-modal distance while enhancing feature alignment. During training, both losses were backpropagated with weighted optimization to balance classification accuracy and multimodal coherence. Training protocols are detailed in Method S3.

#### Reader study

In the human-machine adversarial cohort, the human raters were stratified into two groups: ‌junior otolaryngologists‌ (*n* = 5, ≥3 years of clinical experience in otolaryngology) and ‌senior otolaryngologists‌ (*n* = 3, >8 years of specialized experience in otolaryngology). Each evaluator independently reviewed anonymized medical histories and laryngoscopic images, which were identical to the test cohort’s data composition in terms of lesion characteristics and clinical context. To mitigate observational bias, all identifiers (e.g., patient demographics, treatment history, histopathological outcomes) were concealed during the evaluation process. Within the ‌human-machine adversarial cohort‌, evaluators performed a binary diagnostic task, distinguishing vocal cord dysplasia from glottic carcinoma, mirroring the classification objective of the DL models.

To rigorously assess the clinical utility of the DL models, we performed a head-to-head comparison between model-generated predictions and diagnostic judgments made by human raters within the human-machine adversarial cohort. This analysis quantified diagnostic accuracy, sensitivity, and specificity across both groups, establishing a benchmark for DL algorithm against domain expertise. Furthermore, the comparison was repeated following a 2-month washout period to evaluate potential improvements in diagnostic performance among junior otolaryngologists with the assistance of VLMN.

#### Heatmap generation

To enhance the interpretability of the VLMN model’s predictions, we visualized the image modality through ‌Gradient-weighted Class Activation Mapping (Grad-CAM)‌‌.^44^ Heatmaps for the image modality were generated by implementing gradient-weighted activation hooks on targeted convolutional layers within the PyTorch framework, enabling simultaneous capture of forward-pass feature activations and backward-propagation gradients. This approach isolates spatial dependencies between laryngoscopic features and diagnostic predictions while preserving computational efficiency. In addition, we visualized the text modality by calculating attention weight distributions. Heatmaps for the text modality were generated by computing the global attention received by each token.

### Quantification and statistical analysis

Diagnostic performance for deep learning (DL) models and human raters was assessed using ‌receiver operating characteristic (ROC) curves‌ and ‌area under the curve (AUC)‌ analyses. Additional metrics—including sensitivity, specificity, positive predictive value (PPV), negative predictive value (NPV), and accuracy—were computed to provide granularity in performance characterization. Continuous variables with normal distributions are reported as ‌mean ± standard deviation (SD)‌.

AUC comparisons‌ between models and raters were analyzed using the ‌DeLong test‌‌, a nonparametric method optimized for correlated ROC curves. Differences in classification accuracy on the same test cohort were evaluated via ‌McNemar’s test‌‌, which accounts for paired categorical outcomes. Statistical significance was defined at *p* < 0 · 050 (two-tailed). ‌Confusion matrices‌ were employed to visualize diagnostic concordance and error patterns in glottic lesion classification by both DL models and human raters.

Software implementation protocols and justifications for statistical method selection (e.g., DeLong test for AUC robustness, McNemar’s test for matched-pair accuracy comparisons) are detailed in ‌Method S4‌.

## Supplemental information

Document S1. Figures S1–S5, Tables S1–S6, and Method S1–S4
