# Supplementary material for: A deep learning fusion network trained with medical records and laryngoscopic images in the early diagnosis of glottic carcinoma
Source: iScience. 2025 Nov 26;28(12):114231. doi: 10.1016/j.isci.2025.114231 (PMC12752756; doi:10.1016/j.isci.2025.114231)
Supplement: Document S1. Figures S1–S5, Tables S1–S6, and Method S1–S4 [file mmc1.pdf]

iScience, Volume *28*

Supplemental information

A deep learning fusion network trained with medical records and laryngoscopic images in the early diagnosis of glottic carcinoma

Yi Shuai, Yun Li, Zhaohui Jin, Yuanyuan Li, Lin Chen, Wenqing Chen, Zhenqing Chen, Weixiong Chen, Ruxin Wang, Xiaomao Fan, and Wenbin Lei

Supplemental Information

Figure S1. The prompt template of LLaMA.

Figure S2. ROC curve of VLMN model trained on all vs. early-stage cases.

Figure S3. Confusion matrices of VLMN model trained on all vs. early-stage cases.

Figure S4. ROC curve of VLMN model on WLI and NBI images.

Figure S5. Confusion matrices of VLMN model on WLI and NBI images.

Method S1. Data Preprocessing

Method S2. Development of the vision large language model based multimodal fusion network model

Method S3. Model Training

Method S4. Statistical analysis

Table S1. Manufactures of larynscope equipments in the training, validation and test cohorts.

Table S2. Diagnostic performance of VLMN model in the sub-centers of external test cohort.

Table S3. Proportion of WLI and NBI images used in the training, validation and test cohorts.

Table S4. Diagnostic performance of VLMN model trained on all vs. early-stage cases.

Table S5. Diagnostic performance of VLMN model on WLI and NBI images.

Table S6. Significance of group comparisons in the test cohorts based on AUC.

Prompt

<|begin_of_text|><|start_header_id|>system: <|end_header_id|> You're a very skilled doctor.<|eot_id|>
<|start_header_id|>user: <|end_header_id|> The following is the patient's medical history information, please answer the relevant questions based on this information:
{text}
What is the gender of the patient? What is the age of the patient? Does the patient have symptoms of hoarseness or foreign body sensation in the throat? If so, how long have the symptoms been present? Is it continuous or intermittent? Is it repeated? Is there any aggravation? Can it be relieved after hoarseness? Does the patient have symptoms of acid reflux and belching? Does the patient have any symptoms of blood in the sputum? Does the patient have any symptoms of breathing difficulty? Does the patient have symptoms of dysphagia? Has the patient had a laryngoscopy? What did the examination find? Did you find a vocal cord mass? Is the surface of the tumor rough? What is the extent of the invasion? Has the patient had a histopathological examination? What is the result? Or does it suggest (mild/moderate/severe) atypical hyperplasia/carcinoma in situ/invasive carcinoma? Does the patient have any previous diseases of the digestive system such as throat, esophagus, and stomach? Does the patient smoke or drink alcohol, and if so, how often? How long has it been going on? Has the patient experienced any significant weight loss recently? Does the patient have any other discomfort or conditions that need attention?
No additional information is required for the content of the answer, just answer the question, such as:

Male. 45 years old. Hoarseness symptoms, lasting for 2 weeks, repeated, no aggravation, can be relieved after hoarseness. Symptoms of acid reflux and belching. No blood in sputum. You have difficulty breathing. No dysphagia symptoms. No laryngoscopy. No histopathological examination was performed. No digestive disease. No smoking, no alcohol. No significant weight loss. No other discomfort.

<|eot_id|><|start_header_id|>assistant: <|end_header_id|>

Figure S1. The prompt template of LLaMA (Related to STAR Methods)**.**

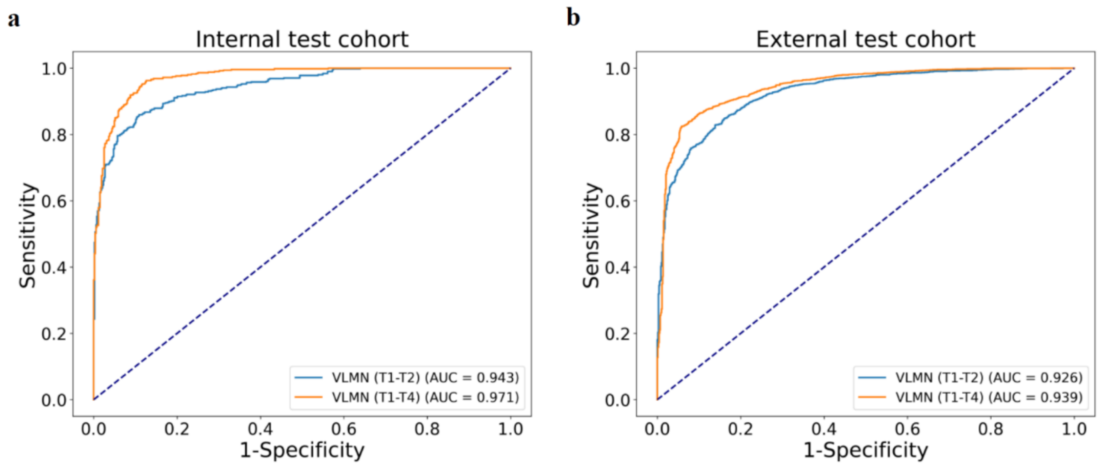

Figure S2. ROC curve of VLMN model trained on all vs. early-stage cases (Related to Figure 1)**.** (a) ROC curves of VLMN model trained on all (T1-T4) vs. early-stage (T1-T2) cases in the internal test cohort. (b) ROC curves of VLMN model trained on all (T1-T4) vs. early-stage (T1-T2) cases in the external test cohort.

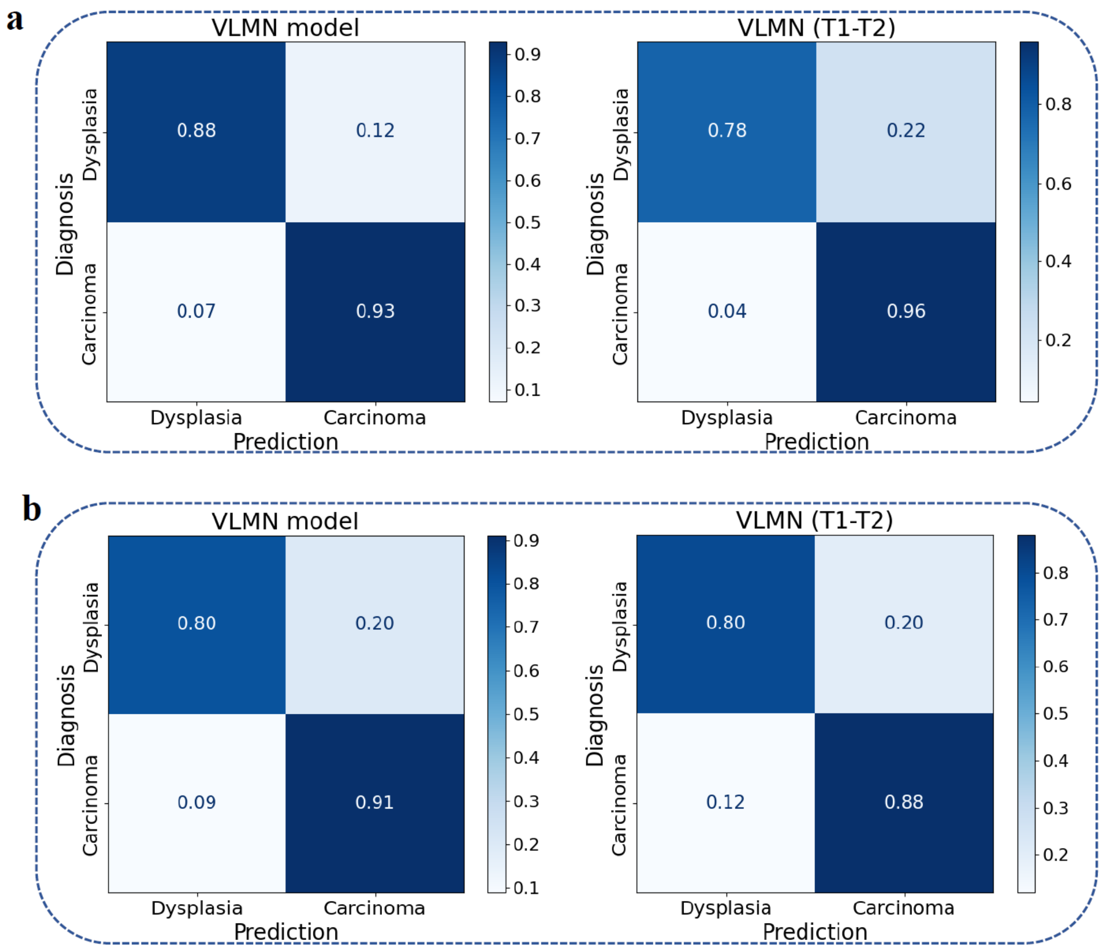

Figure S3. Confusion matrices of VLMN model trained on all vs. early-stage cases (Related to Figure 2)**.**

(a) Confusion matrices of VLMN model trained on all (T1-T4) vs. early-stage (T1-T2) cases in the internal test cohort. (b) Confusion matrices of VLMN model trained on all (T1-T4) vs. early-stage (T1-T2) cases in the external test cohort.

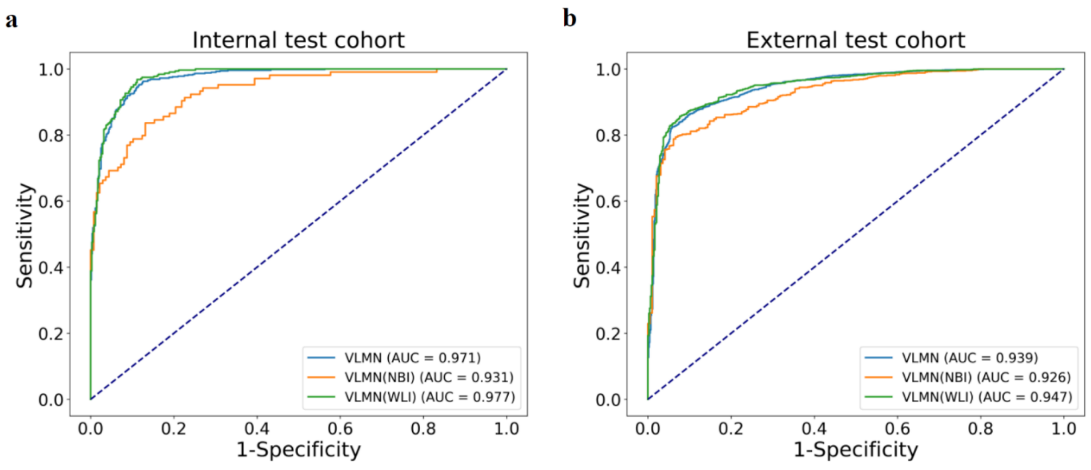

Figure S4. ROC curve of VLMN model on WLI and NBI images (Related to Figure 1)**.**
(a) ROC curves of VLMN model with all enrolled images, WLI images, and NBI images in the internal test cohort. (b) ROC curves of VLMN model with all enrolled images, WLI images, and NBI images in the external test cohort.

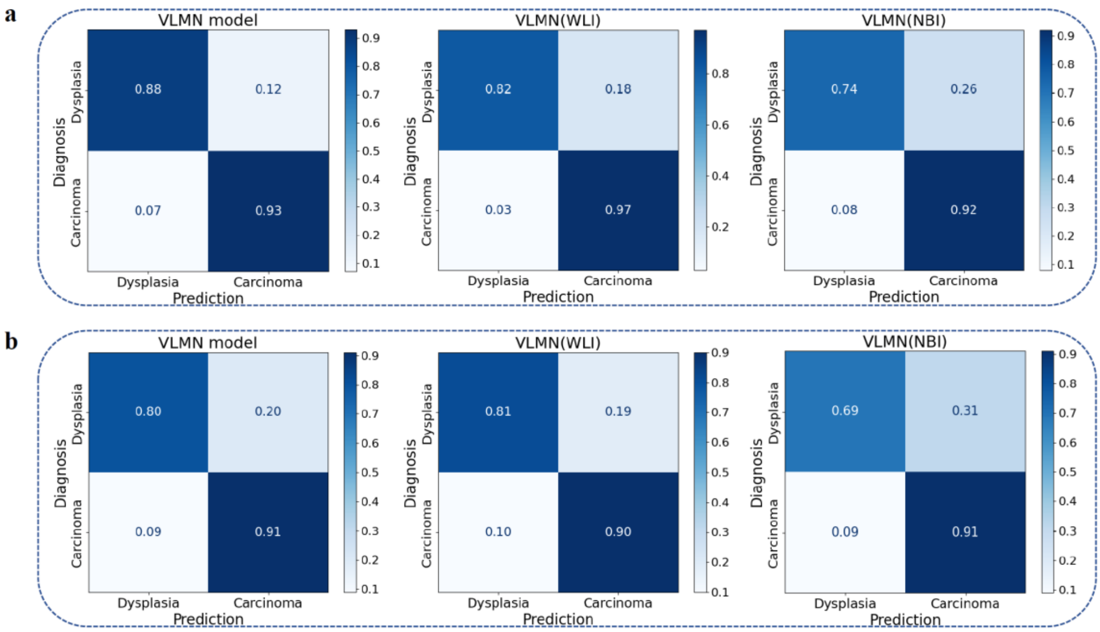

Figure S5. Confusion matrices of VLMN model on WLI and NBI images (Related to Figure 2)**.** (a) Confusion matrices of VLMN model with all enrolled images, WLI images, and NBI images in the internal test cohort. (b) Confusion matrices of VLMN model with all enrolled images, WLI images, and NBI images in the external test cohort.

Method (Related to STAR Methods)

S1. Data Preprocessing

1.1 Prompt Selection

In our study, the prompts were selected based on their established relevance to the clinical evaluation of laryngeal cancer, in accordance with a reasoning process that reflects routine clinical history-taking. For example:

(1) Gender and age are well-known epidemiological risk factors, with laryngeal carcinoma exhibiting a markedly higher incidence in older males.[1,2]

(2) Symptom characteristics and duration, such as persistent hoarseness (>2 weeks) or progressive throat discomfort, are classical clinical indicators of glottic malignancy.

(3) Accompanying symptoms (e.g., dyspnea, dysphagia, blood-streaked sputum) are often associated with more advanced disease.

(4) Weight loss may reflect cancer-related cachexia.

(5) Smoking and alcohol use are long-established etiological risk factors for laryngeal cancer.[2,3]

(6) Gastroesophageal reflux has been increasingly recognized as a potential contributing factor in laryngeal carcinogenesis.[4,5]

(7) Findings from external or outpatient examinations (e.g., previous laryngoscopy or pathology) may also provide valuable diagnostic guidance at the time of presentation.

1.2 Sentence-Level Report Data Extraction

In the text processing section, considering that the original data is an Excel file with a large amount of redundant information, we specifically extracted the patient's age, gender, medical history, current illness history, and personal history. After extracting this data, we applied a fine-grained processing approach to standardize the text. First, under the guidance of professional otolaryngologists, we constructed targeted prompts to standardize the report data and extract diagnosis-related sentence-level reports, thereby improving the model's diagnostic capability. Then, we utilized the LLaMA large language model to automatically extract sentence-level reports from the original diagnostic report,[6] based on the prompts composed of these diagnostic standards, filtering out irrelevant information (the prompt template is illustrated in Figure S1). Once we obtained the sentence-level reports, since the subsequent text feature extraction module uses the pre-trained LLaMA3 model for text feature extraction, we directly used LLaMA3's tokenizer to process the reports for easier extraction of text modality features in the next stage.

1.3 Image Processing

In the image processing section, since we used the pre-trained Vision Transformer (ViT) model for feature extraction in the image feature extraction module,[7] we directly utilized the corresponding processor from the Hugging Face transformers library to preprocess the laryngoscope images. Specifically, in the image preprocessing process of the ViT model, the input image first needs to be uniformly scaled to a fixed size (224×224 in this model), and the image is subsequently normalized. Subsequently, the image is divided into small non-overlapping patches (patches), each patch is usually 16×16 pixels, and a total of 196 patches are obtained for the 224×224 image. each patch is spread into a one-dimensional vector (768 dimensions), which is mapped to a uniform embedding space through a linear layer. Then, in order to inject position information, a learnable position code corresponding to the number of patches is added. A [CLS] token can also be inserted before all patch embedding for the final image classification representation. Finally, these composed token sequences are fed into the ViT backbone Transformer network for feature extraction and subsequent processing.

1.4 Oversampling for class Imbalance

To address the serious imbalance in the number of benign and malignant cases in the dataset, we introduced an oversampling strategy during the model training phase. Specifically, we conducted random repeated sampling on a small number of benign samples in the training set to minimize the gap between them and malignant samples during the training process, thus constructing a more balanced data distribution. This method avoids the model being overly biased towards the majority class (malignant) during training, and improves its ability to recognize minority classes (benign). At the same time, to ensure the objectivity of model evaluation, we only perform oversampling operations on the training set, while keeping the original distribution unchanged on the validation and testing sets to reflect the true clinical data features and provide more clinically meaningful performance evaluations. This strategy effectively reduces the bias risk caused by category imbalance and enhances the robustness and practicality of the model.

S2. Development of the vision large language model based multimodal fusion network model

2.1 Laryngoscope Image Encoder

In the laryngoscope image encoder module, we used a pre-trained Vision Transformer (ViT) model to extract features from the laryngoscope images. Specifically, the entire patch sequence obtained above is fed into a Transformer encoder with multiple stacked layers, each layer consisting of a multi head self attention mechanism and a feedforward network. Finally, we can extract the representation corresponding to the [CLS] token from the output sequence of the Transformer as the visual feature vector of the entire image. Formally, let $f_{enc}(\cdot)$ denote the image encoder. Given an image x_i, the image feature v_i can be obtained as follows:

$$v_i = f_{enc}(x_i\,;\,\theta_{enc})$$

where θ_{enc} represents the parameters of the image encoder. It is worth noting that we directly used the pre-trained weights from the image encoder part of BLIP-2 as the parameters for our laryngoscope image encoder,[8] because extensive research has demonstrated BLIP-2's effectiveness in downstream tasks.[9,10]

2.2 Text Encoder

For the text modality, we adopted the LLaMA3 large language model, which is known for its powerful language processing abilities and its capacity to handle long texts. Compared to other large language models, LLaMA3 performs exceptionally well in processing clinical reports. Formally, let $f_{Llama3}(\cdot)$ represent the text encoder. Given a medical record r_i, the encoder can be represented as:

$$t_i = f_{Llama3}(r_i\,;\,\theta_l)$$

where t_i represents the text embedding vector generated by the text encoder, and θ_l denotes the parameters of the encoder. It is important to note that our text data is in Chinese. However, the original LLaMA3 model's ability to handle Chinese data is weaker than its ability to process English. Therefore, we used the pre-trained LLaMA3 model weights that were trained on Chinese datasets to process our data.[11]

2.3 Multimodal Feature Fusion Network and Classifier

To fuse the image and text modalities, we introduce a multimodal feature fusion network. Specifically, we first use vision embedding $f_{ve}(\cdot)$ and text embedding $f_{te}(\cdot)$ to map the image and text embedding vectors v_i and t_i to a unified representation space, as follows:

$$v_i' = f_{ve}(v_i\,;\,\theta_{ve})$$

$$t_i' = f_{te}(t_i\,;\,\theta_{te})$$

where v_i' and t_i' represent the transformed image and text features, and θ_{ve} and θ_{te} are the parameters of $f_{ve}(\cdot)$ and $f_{te}(\cdot)$, respectively. Additionally, we apply L2 normalization to these two features, obtaining:

$$v_i'' = \frac{v_i'}{||v_i'||_2} \quad \text{and} \quad t_i'' = \frac{t_i'}{||t_i'||_2}$$

Finally, we concatenate the normalized features v_i'' and t_i'' to form the visual-language joint feature g_i, as follows:

$$g_i = \text{Concat}(v_i''\,,\,t_i'')$$

We then pass the visual-language joint feature g_i to the classifier f_{fc}, which is defined as:

$$\hat{y_i} = f_{fc}\,(g_i\,,\,\theta_{fc})$$

where θ_{fc} represents the parameters of the classifier, and $\hat{y_i}$ is the predicted probability distribution of the model.

It is worth noting that both the vision embedding and text embedding use average pooling, linear dimensionality reduction (reduced to 2048 dimensions), and ReLU activation functions for the mapping

operation.

S3. Model Training

3.1 Loss Function

In the loss function section, we use a weighted combination of cross-entropy loss and contrastive loss for backpropagation. The cross-entropy loss is denoted as L_{ce} and the contrastive loss is denoted as L_{ct}. Their expressions are as follows:

$$L_{CE} = -\sum_{i=1}^{C} y_i \log(\hat{y_i})$$

$$L_{CT} = -\log \frac{\exp(sim(z_i, z_j)/\tau)}{\sum_{k=1\,[k\neq i]}^{2N} \exp(sim(z_i, z_k)/\tau)}$$

In L_{ce}, C is the number of classes, and y_i represents the one-hot encoded true labels. In L_{ct}, $sim(\cdot)$ represents the cosine similarity, z_i and z_j are the positive sample pairs, τ is the temperature parameter, and N is the number of negative samples. The final loss function is represented as:

$$L_{loss} = L_{ce} + c * L_{ct}$$

where L_{loss} is the final loss value, and c = 0.1.

3.2 Other Parameters and Equipment Information

All experiments were conducted on a dedicated server equipped with four NVIDIA A6000 GPUs, totaling 196GB of GPU memory. The system runs Ubuntu 20.04.5 LTS and was implemented using Pytorch 3.9.0 and Scikit-learn 1.26.3. In this study, we used the AdamW optimizer to optimize the VLMN model, with an initial learning rate of 0.00001, and employed a warm-up and cosine learning rate schedule to dynamically control the learning rate. The training of VLMN was performed for a total of 10 epochs.

S4. Statistical analysis

We chose the statistical methods for the following reasons: the DeLong test was used to compare the area under the receiver operating characteristic curves (AUCs) between two models, as it provides a non-parametric method for evaluating differences in discriminatory performance while accounting for paired predictions in the same cohort. This test is statistically rigorous for correlated data and avoids parametric assumptions about score distributions. The McNemar's test was selected to assess the statistical significance of differences in classification accuracy between the VLMN model and human raters. This test demonstrates critical advantages in comparing the diagnostic accuracy of two paired tests, effectively accounting for within-subject correlation and minimizing bias from inter-individual variability.

The Statistical analyses and visualizations were performed with Python (version 3.9.0, Python Software

Foundation, State of Delaware, USA), Python module scipy (version 1.13.1; https://github.com/scipy/scipy/releases/tag/v1.13.1), pandas (version 2.2.3; https://github.com/pandas-dev/pandas/releases/tag/v2.2.3), matplotlib (version 3.9.2; https://github.com/matplotlib/matplotlib/releases/tag/v3.9.2), scikit-learn (version 1.5.2; https://github.com/scikit-learn/scikit-learn/releases/tag/1.5.2), and numpy (version 1.26.3, open source Python programming language).

Reference

1. Steuer, C. E., El-Deiry, M., Parks, J. R., Higgins, K. A., & Saba, N. F. (2017). An update on larynx cancer. *CA: A Cancer Journal for Clinicians*, *67*(1), 31–50. https://doi.org/10.3322/caac.21386

2. Huang, J., Chan, S. C., Ko, S., Lok, V., Zhang, L., Lin, X., Lucero-Prisno, D. E., Xu, W., Zheng, Z.-J., Elcarte, E., et al. (2024). Updated disease distributions, risk factors, and trends of laryngeal cancer: A global analysis of cancer registries. *International Journal of Surgery (London, England)*, *110*(2), 810–819. https://doi.org/10.1097/JS9.0000000000000902

3. Liberale, C., Soloperto, D., Marchioni, A., Monzani, D., & Sacchetto, L. (2023). Updates on Larynx Cancer: Risk Factors and Oncogenesis. *International Journal of Molecular Sciences*, *24*(16), 12913. https://doi.org/10.3390/ijms241612913

4. Parsel, S. M., Wu, E. L., Riley, C. A., & McCoul, E. D. (2019). Gastroesophageal and Laryngopharyngeal Reflux Associated With Laryngeal Malignancy: A Systematic Review and Meta-analysis. *Clinical Gastroenterology and Hepatology: The Official Clinical Practice Journal of the American Gastroenterological Association*, *17*(7), 1253-1264.e5. https://doi.org/10.1016/j.cgh.2018.10.028

5. Eells, A. C., Mackintosh, C., Marks, L., & Marino, M. J. (2020). Gastroesophageal reflux disease and head and neck cancers: A systematic review and meta-analysis. *American Journal of Otolaryngology*, *41*(6), 102653. https://doi.org/10.1016/j.amjoto.2020.102653

6. Grattafiori, A., Dubey, A., Jauhri, A., Pandey, A., Kadian, A., Al-Dahle, A., Letman, A., Mathur, A., Schelten, A., Vaughan, A., et al. (2024). *The Llama 3 Herd of Models* (No. arXiv:2407.21783). arXiv. https://doi.org/10.48550/arXiv.2407.21783

7. Dosovitskiy, A., Beyer, L., Kolesnikov, A., Weissenborn, D., Zhai, X., Unterthiner, T., Dehghani, M., Minderer, M., Heigold, G., Gelly, S., et al. (2021). *An Image is Worth 16x16 Words: Transformers for Image Recognition at Scale* (No. arXiv:2010.11929). arXiv. https://doi.org/10.48550/arXiv.2010.11929

8. Li, J., Li, D., Savarese, S., & Hoi, S. (2023). *BLIP-2: Bootstrapping Language-Image Pre-training with Frozen Image Encoders and Large Language Models* (No. arXiv:2301.12597). arXiv. https://doi.org/10.48550/arXiv.2301.12597

9. Zhu, D., Chen, J., Haydarov, K., Shen, X., Zhang, W., & Elhoseiny, M. (2023). *ChatGPT Asks, BLIP-2 Answers: Automatic Questioning Towards Enriched Visual Descriptions* (No. arXiv:2303.06594). arXiv. https://doi.org/10.48550/arXiv.2303.06594

10. Nguyen, T., Gadre, S. Y., Ilharco, G., Oh, S., & Schmidt, L. (2023). *Improving Multimodal Datasets with Image Captioning* (No. arXiv:2307.10350). arXiv. https://doi.org/10.48550/arXiv.2307.10350

11. Cui, Y., Yang, Z., & Yao, X. (2024). *Efficient and Effective Text Encoding for Chinese LLaMA and Alpaca* (No. arXiv:2304.08177). arXiv. https://doi.org/10.48550/arXiv.2304.08177

Table S1. Manufactures of larynscope equipments in the training, validation and test cohorts (Related to STAR Methods)**.**

Cohort	Laryngoscope model	Manufacturers	FOV	DOF	Working length	Angle range	Total length	NBI
**Training, validation and internal test cohorts**	ENF-VT2	Olympus, Tokyo, Japan	90°	2-40mm	365mm	130° upward, 130° downward	635mm	Yes
	ENF-VT3	Olympus, Tokyo, Japan	90°	2-50mm	365mm	130° upward, 130° downward, 70° to the right, 70° to the left	645mm	Yes
	ENF-VH	Olympus, Tokyo, Japan	110°	5.0-50mm	300mm	130° upward, 130° downward	510mm	Yes
	ENF-V3	Olympus, Tokyo, Japan	90°	3.5-50mm	300mm	130° upward, 130° downward	510mm	Yes
**External test cohort**								
FPHFS	ENF-VH	Olympus, Tokyo, Japan	110°	5.0-50mm	300mm	130° upward, 130° downward	510mm	Yes
	ENF-V3	Olympus, Tokyo, Japan	90°	3.5-50mm	300mm	130° upward, 130° downward	510mm	Yes
FPHZQ	ENF-V2	Olympus, Tokyo, Japan	90°	5.0-50mm	300mm	130° upward, 130° downward	510mm	No
	ENF-V3	Olympus, Tokyo, Japan	90°	3.5-50mm	300mm	130° upward, 130° downward	510mm	Yes

Abbreviations: FOV, Field of View; DOF, Depth of Field; NBI, Narrow Band Imaging; FPHFS, the First People's Hospital of Foshan; FPHZQ, the First People's Hospital of Zhaoqing.

Table S2. Diagnostic performance of VLMN model in the sub-centers of external test cohort (Related to Table 2)**.**

**Ratings**	**Accuracy (95% CI)**	**AUC (95% CI)**	**Sensitivity (95% CI)**	**Specificity (95% CI)**	**PPV (95% CI)**	**NPV (95% CI)**
**VLMN**						
FPHFS	0.905 (0.897-0.913)	0.963 (0.960-0.966)	0.960 (0.949-0.970)	0.694 (0.645-0.743)	0.924 (0.903-0.945)	0.812 (0.759-0.865)
FPHZQ	0.708 (0.684-0.733)	0.778 (0.771-0.785)	0.662 (0.627-0.697)	0.850 (0.791-0.910)	0.920 (0.864-0.975)	0.474 (0.366-0.582)

Abbreviations: VLMN, vision large language model based multimodal fusion network; FPHFS, The First People's Hospital of Foshan; FPHZQ, The First People's Hospital of Zhaoqing; AUC, area under the receiver operating characteristic curve; PPV, positive predictive value; NPV, negative predictive value; CI, confidence interval.

Table S3. Proportion of WLI and NBI images used in the training, validation and test cohorts (Related to Table 1)**.**

	Training cohort	Validation cohort	Internal test cohort	External test cohort	
				**FPHFS**	**FPHZQ**
**WLI+NBI** (n)	7780	1135	980	3238	390
**WLI** (n, %)	5886 (75.7%)	885 (78.0%)	718 (73.3%)	2012 (62.1%)	288 (73.8%)
**NBI** (n, %)	1894 (24.3%)	250 (22.0%)	262 (26.7%)	1226 (37.9%)	102 (26.2%)
**Mean image count** (n)	17	16	15	37	8

Abbreviations: WLI, White Light Imaging; NBI, Narrow Band Imaging; FPHFS, the First People's Hospital of Foshan; FPHZQ, the First People's Hospital of Zhaoqing.

Table S4 Diagnostic performance of VLMN model trained on all vs. early-stage cases (Related to Table 2)**.**

Ratings	Accuracy (95% CI)	AUC (95% CI)	Sensitivity (95% CI)	Specificity (95% CI)	PPV (95% CI)	NPV (95% CI)
**Internal test cohort**						
VLMN[a]	0.901 (0.888-0.914)	0.971 (0.968-0.974)	0.858 (0.824-0.891)	0.956 (0.931-0.981)	0.959 (0.932-0.985)	0.845 (0.797-0.892)
VLMN(T1–T2) [b]	0.868 (0.818-0.917)	0.957 (0.946-0.969)	0.950 (0.893-1.000)	0.782 (0.670-0.895)	0.823 (0.753-0.892)	0.942 (0.885-0.996)
**External test cohort**						
VLMN[a]	0.884 (0.875-0.893)	0.939 (0.933-0.944)	0.945 (0.930-0.960)	0.682 (0.638-0.726)	0.908 (0.884-0.933)	0.784 (0.710-0.858)
VLMN(T1–T2) [b]	0.865 (0.825-0.906)	0.922 (0.878-0.967)	0.880 (0.814-0.946)	0.807(0.736-0.878)	0.944 (0.935-0.952)	0.650(0.622-0.679)

Abbreviations: VLMN[a], the model trained on all enrolled cases, including T3–T4 glottic carcinoma; VLMN (T1–T2) [b], the model trained exclusively on early-stage (T1–T2) cases; AUC, area under the receiver operating characteristic curve; PPV, positive predictive value; NPV, negative predictive value; CI, confidence interval.

Table S5 Diagnostic performance of VLMN model on WLI and NBI images (Related to Table 2)**.**

Ratings	Accuracy (95% CI)	AUC (95% CI)	Sensitivity (95% CI)	Specificity (95% CI)	PPV (95% CI)	NPV (95% CI)
**Internal test cohort**						
VLMN[a]	0.901 (0.888-0.914)	0.971 (0.968-0.974)	0.858 (0.824-0.891)	0.956 (0.931-0.981)	0.959 (0.932-0.985)	0.845 (0.797-0.892)
VLMN (WLI)[b]	0.894 (0.861-0.926)	0.976 (0.971-0.980)	0.974 (0.946-1.000)	0.822 (0.741-0.904)	0.833 (0.773-0.894)	0.974 (0.948-0.999)
VLMN (NBI)[c]	0.820 (0.769-0.870)	0.935 (0.921-0.949)	0.919 (0.865-0.974)	0.744 (0.620-0.869)	0.740 (0.645-0.836)	0.927 (0.894-0.960)
**External test cohort**						
VLMN[a]	0.884 (0.875-0.893)	0.939 (0.933-0.944)	0.945 (0.930-0.960)	0.682 (0.638-0.726)	0.908 (0.884-0.933)	0.784 (0.710-0.858)
VLMN (WLI)[b]	0.874 (0.865-0.883)	0.941 (0.934-0.948)	0.905 (0.838-0.972)	0.808 (0.640-0.976)	0.911 (0.844-0.978)	0.810 (0.728-0.892)
VLMN (NBI)[c]	0.862 (0.856-0.868)	0.934 (0.926-0.942)	0.914 (0.856-0.972)	0.690 (0.508-0.874)	0.910 (0.866-0.954)	0.731 (0.618-0.843)

Abbreviations: VLMN[a], the diagnostic performance of VLMN model on all enrolled laryngoscopic images; VLMN (WLI)[b], the diagnostic performance of VLMN model on WLI images; VLMN (NBI)[c], the diagnostic performance of VLMN model on NBI images; AUC, area under the receiver operating characteristic curve; PPV, positive predictive value; NPV, negative predictive value; CI, confidence interval.

Table S6. Significance of group comparisons in the test cohorts based on AUC (Related to Table 2)**.**

P value	Internal test cohort			External test cohort		
	**VLMN model**	**Image-based monomodal model**	**Text-based monomodal model**	**VLMN model**	**Image-based monomodal model**	**Text-based monomodal model**
**Internal test cohort**						
VLMN model	NA	<0.0001	0.035	NA	NA	NA
Image-based monomodal model	<0.0001	NA	0.40	NA	NA	NA
Text-based monomodal model	0.035	0.40	NA	NA	NA	NA
**External test cohort**						
VLMN model	NA	NA	NA	NA	< 0.0001	0.042
Image-based monomodal model	NA	NA	NA	<0.0001	NA	0.23
Text-based monomodal model	NA	NA	NA	0.042	0.23	NA

Abbreviations: VLMN, vision large language model based multimodal fusion network; AUC, area under the receiver operating characteristic curve; NA, not applicable.
